# Ken Fearon

**DOI:** 10.1002/jcsm.12187

**Published:** 2017-02-27

**Authors:** Richard J.E. Skipworth, James A. Ross

**Affiliations:** ^1^ Clinical SurgeryUniversity of Edinburgh, Royal Infirmary of EdinburghEdinburghEH16 4SA

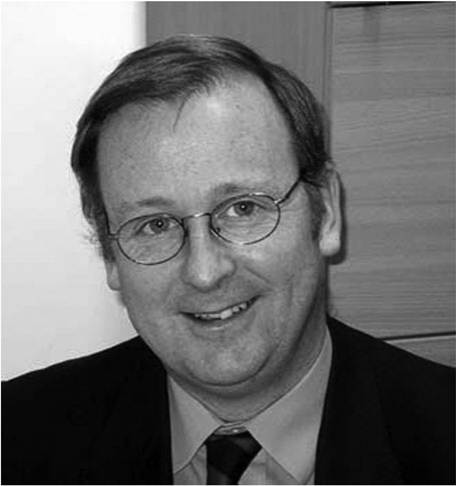

Professor Ken Fearon, who was Professor of Surgical Oncology at the University of Edinburgh, UK, and an Honorary Consultant Colorectal Surgeon at the Western General Hospital, Edinburgh, died suddenly on the 3^rd^ September, 2016, aged 56 years. We remember him here in the pages of the Journal of Cachexia, Sarcopenia and Muscle, as a leading light in the field of cancer cachexia.

## Career

Ken attended St Aloysius’ College, an independent Jesuit school in Glasgow, and he was proud of both his faith and his West of Scotland origins. From the day he entered Glasgow Medical School, it was evident that Ken had a fierce intellect and that he was an academic clinician to watch in the future. He achieved numerous distinctions in medical school, and in his final examinations, he was awarded the Brunton Memorial Prize as the top undergraduate. After qualification and completion of his junior doctor posts, Ken was the Cancer Research Clinical Research Fellow in the department of oncology in Glasgow University from 1983‐1986. It was this period of postgraduate research that ignited and cemented his life‐long interest in cancer cachexia, and which led to publication of his 1987 MD thesis, “Mechanisms and Treatment of Cancer Cachexia”. At this time, little was known of the mechanisms of cancer cachexia, but since then, Ken has been one of the chief driving forces in our improved understanding of this complex, multifactorial syndrome. In 1988, Ken was appointed as lecturer in the Department of Surgery at the University of Edinburgh. He was subsequently promoted to senior lecturer and honorary consultant surgeon at the youthful age of 32 years, before ultimately being appointed as professor of surgical oncology in 1999. Despite his significant scientific and research commitments, Ken was firmly dedicated to his patients and he was always careful to remind his juniors that research was performed in order for their benefit.

## Research interests

In his own words, Ken wrote that his research focus was “a multidisciplinary approach to the understanding of the mediators and mechanisms of wasting in cancer cachexia”, and that he aimed to perform studies in human integrative physiology that “extend from the molecular level to whole body metabolic studies”. This bench‐to‐bedside approach set Ken apart as a unique clinical researcher that could investigate (and understand) effectively the full breadth of the cachexia syndrome. His recent projects concentrated on “better understanding of the pathophysiology of muscle wasting, the development of biomarkers for clinical trials and the possibility that there is a genetic predisposition to wasting”.

In his constant search for an effective therapeutic platform for cachexia, Ken gained a strong reputation as a clinical trials investigator, with an interest in clinical trial design and the development of novel endpoints as outcome measures. He was one of the first researchers to investigate the impact of anti‐inflammatory fish oils on body composition in cancer patients, and, he subsequently went on to perform intervention trials of other agents, including beta‐agonists, myostatin antagonists, and ghrelin analogues. Through such trials, he and others identified that the lack of an agreed, clinically relevant definition of cancer cachexia was a major hindrance to the demonstration of therapeutic efficacy. In answer to this problem, the 2011 Fearon et al publication in Lancet Oncology entitled “Definition and classification of cancer cachexia: an international consensus” is now considered a landmark paper, and is the most cited in this field.

In total, Ken published over 300 peer‐reviewed publications and continues to publish after his death. Over 170 of these papers were focused on cancer cachexia, with the remainder concerning his other passionate research interests of colorectal surgery, surgical nutrition, and, in particular, enhanced recovery after surgery (ERAS).

## Societies and awards

As both a clinician and a researcher, Ken served on many national and international committees. He was Past President of the International Association for Surgical Nutrition and Metabolism; Chairman of the Research Board at the Royal College of Surgeons of Edinburgh; and Chairman of the Board of Directors for the ERAS Society.

Ken deservedly received many awards and lectureships during his career, including the David Cuthbertson Medal from the Nutrition Society of Great Britain and Ireland 1991; the RB Wright Lectureship from Royal College of Physicians and Surgeons of Glasgow 2000; Bengt Ihre Lectureship from the Swedish Surgical Society 2003; Hippocrates Award from the Society on Sarcopenia, Cachexia and Wasting Disorders (SCWD) 2009; the Arvid Wretlind Award from the European Society for Clinical Nutrition and Metabolism 2011; and the Sir William MacEwen Lectureship at the Association of Surgeons of Great Britain and Ireland 2014. He served as a visiting professor at McGill University (Canada) and the Dutch Surgical Society, and he was awarded an honorary doctorate by Orebro University in Sweden. Since his passing, further tributes have been paid in Ken's honour, including the inception of the Ken Fearon oral presentation prize at the ERAS Society UK Conference, and the “Meet the Mentors” session at the excellently titled Ken Fearon Career Café at the Cachexia Conference.

## Teaching and mentorship

Ken was a superlative teacher and mentor to both undergraduate students and junior surgeons. He supervised a total of 13 postgraduate doctoral fellows and 2 Masters students during his career. Ken (or “Prof” as he was known to his students) delivered his education in a thoughtful and scientifically rigorous manner, but always with humour and sometimes the occasional voice impression! He was a deep well of good career advice, and was always calm and sympathetic in the face of disaster. As a reflection of his mentorship abilities, he was awarded the Role Model in Academic Medicine Award from the British Medical Association in 2006.

## Other interests

Ken was a lover of golf, fine wine, fine art and Italian holidays. He was extremely knowledgable on such subjects and could fondly deliver erudite and ‘off‐the‐cuff’ lectures as required.

In short, Ken was an exceptional person who was able to traverse successfully the worlds of surgery and science. His prodigious research output stemmed, in part, from his ability to forge friendships and collaborations wherever he travelled. He conducted himself and his research with an exceptional human touch, a sense of humour, and insightfulness, and it is for these reasons that so many of his former students and colleagues hold him in such high regard. Those of us who worked closely with Ken find it difficult to believe what has happened, and we are reminded of our conversations with him about family, friends, former research fellows and general academic gossip. When we did speak of work, Ken would unleash his formidable intellect and provide insights into our current efforts with logic, wit and persuasion. We will miss him enormously and we are sure he would have been touched to know so many colleagues thought so highly of him.

He is survived by his wife, Professor Marie Fallon, and their two young children.

